# Challenge of Reducing Perinatal Mortality in Rural Congo: Findings of a Prospective, Population-based Study

**DOI:** 10.3329/jhpn.v29i5.8908

**Published:** 2011-10

**Authors:** Richard M. Matendo, Cyril M. Engmann, John D. Ditekemena, Justin Gado, Antoinette Tshefu, Elizabeth M. McClure, Janet Moore, Marleen Boelaert, Waldemar A. Carlo, Linda L. Wright, Carl L. Bose

**Affiliations:** ^1^Kinshasa School of Public Health, Kinshasa, Democratic Republic of Congo; ^2^Department of Pediatrics, University of North Carolina, Chapel Hill, North Carolina, USA; ^3^Research Triangle Institute, Durham, North Carolina, USA; ^4^Unit of Epidemiology, Prince Leopold Institute of Tropical Medicine, Antwerp, Belgium; ^5^Department of Pediatrics, University of Alabama at Birmingham, Birmingham, Alabama, USA,; ^6^Eunice K Shriver National Institute of Child Health and Human Development, National Institutes of Health, Bethesda, MD, USA

**Keywords:** Neonatal mortality, Observational studies, Perinatal mortality, Population-based studies, Prospective studies, Stillbirths, Congo

## Abstract

Each year, an estimated six million perinatal deaths occur worldwide, and 98% of these deaths occur in lowand middle-income countries. These estimates are based on surveys in both urban and rural areas, and they may underrepresent the problem in rural areas. This study was conducted to quantify perinatal mortality, to identify the associated risk factors, and to determine the most common causes of early neonatal death in a rural area of the Democratic Republic of the Congo (DRC). Data were collected on 1,892 births. Risk factors associated with perinatal deaths were identified using multivariate analysis with logistic regression models. Causes of early neonatal deaths were determined by physician-review of information describing death. The perinatal mortality rate was 61 per 1,000 births; the stillbirth rate was 30 per 1,000 births; and the early neonatal death rate was 32 per 1,000 livebirths. Clinically-relevant factors independently associated with perinatal death included: low birthweight [odds ratio (OR)=13.51, 95% confidence interval (CI) 7.82-23.35], breech presentation (OR)=12.41; 95% CI 4.62-33.33), lack of prenatal care (OR=2.70, 95% CI 1.81-4.02), and parity greater than 4 (OR=1.93 95% CI 1.11-3.37). Over one-half of early neonatal deaths (n=37) occurred during the first two postnatal days, and the most common causes were low birthweight/prematurity (47%), asphyxia (34%), and infection (8%). The high perinatal mortality rate in rural communities in the DRC, approximately one-half of which is attributable to early neonatal death, may be modifiable. Specifically, deaths due to breech presentation, the second most common risk factor, may be reduced by making available emergency obstetric care. Most neonatal deaths occur soon after birth, and nearly three-quarters are caused by low birthweight/prematurity or asphyxia. Neonatal mortality might be reduced by targeting interventions to improve neonatal resuscitation and care of larger preterm infants.

## INTRODUCTION

Each year, an estimated six million perinatal deaths (the combination of stillbirths and early neonatal deaths) occur worldwide, representing 4% of the global burden of disease (1-3), and 98% of these deaths occur in lowand middle-income countries (LMICs). Perinatal deaths are the leading burden of disease among children aged less than five years worldwide (4). In this age-group, perinatal deaths cause twice as many deaths as malaria and HIV/AIDS combined (3-5). Because of the contribution of perinatal mortality to child mortality, unless effective interventions aimed at reducing perinatal mortality are implemented, achieving the Millennium Development Goal 4 of reducing under-five mortality by two-thirds in 2015 will be impossible (6).

The health burden of perinatal mortality is particularly high in the Democratic Republic of the Congo (DRC). Based on estimates from survey data, the DRC has the seventh highest neonatal mortality rate (NMR) in the world and the third highest in Africa (2). Since rural populations tend to be underrepresented in survey data, these figures may underestimate the magnitude of the problem (7). In addition, surveys describing perinatal mortality in the DRC have not typically analyzed the associations between risk factors and mortality. These analyses are important tools for developing strategies to reduce mortality.

We sought to determine the perinatal mortality rate (PMR) in a population-based study in a typical rural province in the DRC. We also examined the risk factors for perinatal mortality and determined the most common causes of early neonatal deaths to identify the potential strategies for reducing mortality.

## MATERIALS AND METHODS

### Sudy setting

The DRC is the fourth most populous country in Africa. Despite vast natural resources, unstable political leadership compounded by wars and civil unrest have resulted in the DRC ranking in the bottom 10 countries in the world on the human development index (8). The rural province of Equateur, where this study was conducted, has a health system characterized by limited resources and barriers to accessing healthcare. The primary site of care is the health centre where there is usually no electricity or plumbing. The health centres typically have primitive laboratory facilities and inadequately-stocked pharmacies. Access is limited by long distances between many villages and health centres and a total lack of public transportation. Since most services must be purchased by families and patients, poverty also limits access to healthcare. The population in this province is among the poorest in the country. One third of the population is in the lowest economic quintile in the country. These factors result in a population with poor health outcomes. The acute malnutrition rate is 11%; severe stunting occurs in nearly one-third of young children (9). The mortality rate is 3.9 deaths per 10,000 inhabitants per day among children aged 6-59 months (De Radigues X and Alberti K. Personal communication, 2003).

### Subjects and study design

This prospective, population-based, observational study was nested within a cluster-randomized controlled trial—the FIRST BREATH Trial—, conducted by the Eunice Kennedy Shriver National Institute of Child Health and Human Development's Global Network for Women's and Children's Health Research (10). The FIRST BREATH Trial investigated the benefits of implementing a package of neonatal care practices in community settings. As part of this study, birth attendants were trained to collect basic data on maternal, foetal and neonatal outcomes, which included demographics, mode of delivery, birthweight, gestational age, need for resuscitation, and details of adverse events. Data for the study reported in this paper were collected during the baseline period of the FIRST BREATH Trial (June-December 2005), after training on infant examination and data-collection but before the first intervention. The FIRST BREATH Trial (NCT00136708) is registered with a clinical trials database (www.ClinicalTrials.gov).

Ten communities participated in the study. Pregnant women were enrolled at their first antenatal visit, which was typically by 24 weeks of gestation, or at the onset of labour for those who did not attend antenatal care. Community Coordinators, specially-trained nurses, oversaw data-collection by birth attendants in the community. After each delivery, the Community Coordinators collected data recorded by the birth attendants. Traditional birth attendants (TBAs) were women who assisted during childbirth. TBAs received variable training, which was usually limited to an apprenticeship with an experienced TBA. Skilled birth attendants were nurses, midwives, or doctors who provided obstetric care. If a delivery was not attended by either a TBA or a skilled birth attendant, data describing the circumstances of birth were collected retros-pectively (usually within one day), and the infant was weighed at this time. We collected data describing maternal factors that might influence the pregnancy outcomes. These factors included those for which data were readily available, likely to be highly reliable, and could be expressed numerically or categorically. Variables included the mother's age, education, and living circumstances and also the pregnancy and intrapartum factors. The places of delivery included hospitals, health centres, homes (including the birth attendant's home), or other (in transit). A woman was considered to have participated in antenatal care if she attended at least one visit with a skilled birth attendant.

The inclusion criteria required birth in a study community and a birthweight of 1,000 g or more. We defined early neonatal death (END) as death of a liveborn infant at or before seven days of life (11). Since times of births were not recorded, the age at death was calculated by subtracting the date of birth from the date of death. A stillbirth was a newborn of at least 1,000 g of birthweight or corresponding to approximately 28 weeks of gestation or more, with no signs of life at birth, i.e. no breathing, no heart rate, and no movement.

To ensure the proper differentiation between a stillbirth and a neonatal death, all the birth attendants were trained to check for foetal and neonatal vital signs by auscultating the abdomen of every pregnant woman before delivery and after delivery by feeling the infant's umbilical cord for a pulse, auscultating the lungs for breath sounds, and assessing for any movement. This training also included the identification of physical findings necessary to differentiate a fresh from a macerated stillbirth. All instructions used a train-the-trainer model outlined previously (7). Birthweights were obtained within 48 hours of delivery using the spring Salter Scales (model 145555) of the United Nations Children's Fund (UNICEF) provided for the study. The gestational age was determined using the mother's last menstrual period, when available.

A single physician assigned a cause for each neonatal death using descriptions of the circumstances of death provided by the Community Coordinators. Clinical data were not collected in a uniform manner; laboratory data, e.g. blood smears or cultures, were rarely available. This assignment was based on the physician's knowledge of diseases in the community, assisted by operational definitions. Birth asphyxia was assigned when death followed the inability to breathe spontaneously or sustain breathing soon after birth (12). Low birthweight (LBW) and prematurity were combined as a potential cause of death and were assigned if mortality occurred in an infant who weighed less than 2,500 g at birth or had the appearance of a premature infant according to the Ballard morphologic criteria (13). Infection was assigned in the following circumstances: maternal infection diagnosed during pregnancy and treated without success, prolonged rupture of the membranes, foul-smelling amniotic fluid, maternal fever during labour, or fever or other signs of newborn infection during the first week of life. These signs included lethargy, rapid breathing, and mottled skin. Neonatal tetanus was assigned if mortality occurred in an infant with a normal abili-ty to suck and cry during the first two days of life but thereafter could not suck normally and became stiff or had jerking of the muscles. Congenital defects were assigned as the cause of death when they were presumed to be incompatible with life.

Data were first collected on paper forms and then entered into an electronic database in Equateur. Digitized data were then transmitted electronically to the Data Coordinating Center (RTI International, Research Triangle Park, NC, USA).

### Statistical methods

Data were analyzed using the SAS software (version 9.2). Factors potentially associated with perinatal mortality were grouped into obstetric and sociodemographic maternal characteristics of the infant, type of birth attendant, location of the delivery, and characteristics. Odds ratios (ORs) and 95% confidence intervals (CIs) were computed using logistic regression models with generalized estimating equations adjusting for cluster to assess the relationship between perinatal mortality and each selected variable. Factors associated with stillbirths and neonatal deaths were not examined independently because of limitations of the sample-size and the relative infrequency of these outcomes. Reference categories were determined for each group. A multivariate logistic regression model with generalized estimating equations adjusting for cluster was used for computing adjusted ORs and 95% CI adjusting for all the variables.

### Ethical aspects

The study was approved by the institutional ethics review committees of the Kinshasa School of Public Health, University of North Carolina, and RTI International. Verbal informed consent was obtained from mothers at the time of enrollment.

## RESULTS

### Study population

**Fig. 1.**
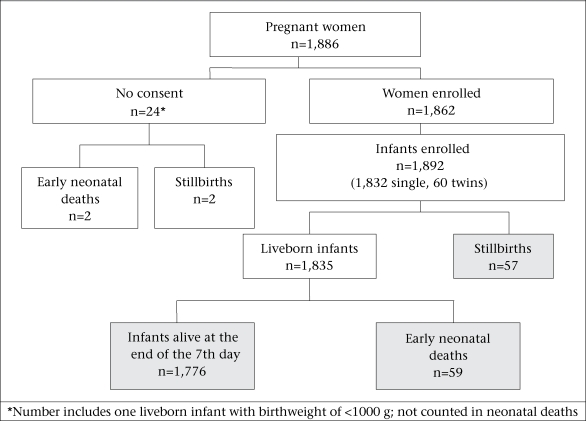
Flow-chart of study participants

In total, 1,886 pregnant women delivered in the study communities during the data-collection period, and 1,862 (99%) consented to participate in the study (Fig. 1). Consent was not obtained for collection of information about 24 pregnancies. One of these pregnancies resulted in the birth of an infant weighing less than 1,000 g at birth; this mother and the infant were excluded from the study. The median age of the mothers was 23 years. Most (98%) women were living with a male partner, and two-thirds had never been to school (Table). Approximately 94% of the women had attended at least one antenatal visit during the current pregnancy.

The 1,862 women delivered 1,892 infants. Of these, 1,835 infants were born alive, 57 were stillborn, and 59 died within seven days after birth. Approximately 80% of the infants were delivered at home, and a similar percentage was delivered by TBAs. Ninety-seven percent of the deliveries were vaginal cephalic deliveries; 2.5% were vaginal breech deliveries, and less than 1% were delivered by caesarean section. Birthweights were normally distributed with a mean weight of 3,069 g [standard deviation (SD) 569 g]; 11% were LBW, and 1.3% were very low-birthweight (VLBW) infants.

Limited information was available about the 23 women for which consent was not obtained. Two delivered in a clinic, and 20 delivered either in their home or in the home of a birth attendant; the place of birth of one is not known. Two were delivered by a nurse or a nurse-midwife, 11 by TBAs, and the remainder by other individuals. Of the 23 infants, two were reported stillbirths, and two died shortly after birth.

### Characteristics of perinatal deaths

**Fig. 2.**
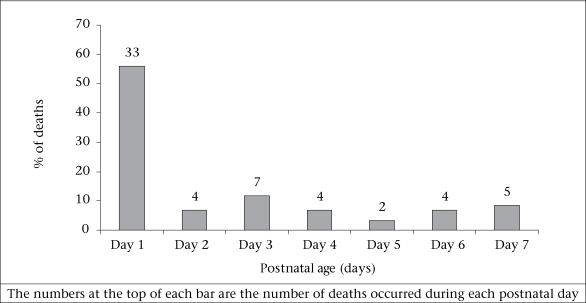
Age of infants at the time of early neonatal death

The rate of stillbirth was 30 per 1,000 births (95% CI 22-38]. Thirty-seven of the 57 stillbirths were fresh. The remaining stillbirths were macerated. Forty stillbirths occurred in the homes, and the remaining stillbirths occurred either in health centers or in hospitals. Twenty-eight of the stillbirths were LBW, and approximately thirteen were VLBW. The END rate was 32 per 1,000 livebirths (95% CI 24-40). Thirty-three of the 59 ENDs occurred on the day of birth or during the following day, and almost two-thirds during the first three days following birth (Fig. 2). Of the 33 infants who died during the first day following birth, 21 were LBW, of whom seven were VLBW. The PMR was 61 per 1,000 births (95% CI 51-72).

### Causes of early neonatal deaths

LBW/prematurity, asphyxia, and infection were assigned as the causes of 47%, 34%, and 8% of ENDs respectively. Two infants died due to neonatal tetanus, one due to complications of spina bifida, and three ENDs were of unknown cause. Three of the five ENDs attributed to infection occurred between the third and the sixth day following birth.

### Predictors of perinatal deaths

LBW and vaginal breech delivery independently conferred a greater than 10-fold increase in the risk of perinatal death. The odds for perinatal death increased seven-fold if the mother was not living with a male partner, and nearly three-fold if the mother did not receive any antenatal care. Parity greater than four increased risk, and multiple gestation birth decreased risk.

## DISCUSSION

In this population-based study, we characterized perinatal mortality in a rural province of the DRC. The PMR was 61 per 1,000 birth**s**. LBW, breech vaginal delivery, mothers not living with a male partner, lack of antenatal care, and parity greater than four all independently conferred an increased risk of perinatal death. Approximately 90% of the ENDs were attributed to asphyxia, LBW/prematurity, and infections, and nearly one-half occurred during the first day after birth.

**Table.** Analysis of risk factors for perinatal mortality

Risk factor	All births*	Perinatal deaths		Crude OR (95% CI)	Adjusted OR (95% CI)
		No.	%		
Place of birth					
Home	1,481	82	5.5	0.65 (0.35-1.21)	0.51 (0.25-1.04)
Health centre/hospital	411	34	8.3	1.00 (-)	1.00 (-)
Birth attendant					
Traditional birth attendant	1,498	82	5.5	0.62 (0.32-1.18)	1.71 (0.74-3.97)
Nurse/midwife/physician	337	29	8.6	1.00 (-)	1.00 (-)
Other**	57	5	8.8	1.02 (0.39-2.65)	2.54 (0.59-10.97)
Age (years) of mothers at delivery					
14-19	399	28	7.0	1.20 (0.72-2.00)	0.92 (0.38-2.23)
20-29	848	50	5.9	1.00 (-)	1.00 (-)
30-39	421	22	5.2	0.88 (0.63-1.23)	0.59 (0.29-1.21)
40-49	41	6	14.6	2.74 (1.05-7.10)	1.95 (0.43-8.90)
Mother living with a male partner					
Yes	1,860	109	5.9	1.00 (-)	1.00 (-)
No	32	7	21.9	4.50 (1.80-11.24)	7.01 (2.32-21.23)
Education of mothers					
No schooling	1,224	84	6.9	2.24 (0.48-10.34)	2.91 (0.39-21.40)
Primary	571	29	5.1	1.62 (0.38-6.88)	2.01 (0.41-9.91)
Secondary/superior/ university	94	3	3.2	1.00 (-)	1.00 (-)
Antenatal care					
Yes	1,780	98	5.5	1.00 (-)	1.00 (-)
No	112	18	16.1	3.29 (2.38-4.55)	2.70 (1.81-4.02)
Parity of mother					
0	384	29	7.6	1.55 (0.98-2.43)	1.04 (0.44-2.44)
1-4	1,096	55	5.0	1.00 (-)	1.00 (-)
5+	411	32	7.8	1.60 (1.10-2.31)	1.93 (1.11-3.37)
No. of foetuses					
Single	1,832	104	5.7	1.00 (-)	1.00 (-)
Multiple	60	12	20.0	4.15 (2.23-7.74)	0.31 (0.13-0.76)
Mode of delivery					
Vaginal, cephalic	1,832	91	5.0	1.00 (-)	1.00 (-)
Vaginal, breech	47	24	51.1	19.96 (12.94-30.80)	12.41 (4.62-33.33)
Caesarean section	3	1	33.3	9.57 (0.50-181.54)	22.07 (0.14-3,596.69)
Sex of baby					
Male	963	66	6.9	1.28 (0.75-2.17)	1.52 (0.84-2.73)
Female	919	50	5.4	1.00 (-)	1.00 (-)
Birthweight (g)					
1,000-2,499	207	63	30.4	14.38 (9.52-21.74)	13.51 (7.82-23.35)
2,500-4,000	1,626	48	3.0	1.00 (-)	1.00 (-)
4,001-5,000	45	2	4.4	1.53 (0.48-4.83)	1.28 (0.30-5.39)

*The maximum number of mothers or infants for each category was the number for which data describing risk factor were available;

**Other=Unattended or attended by a family member only;

CI=Confidence interval;

OR=Odds ratio

The PMR observed in our study was lower than the WHO-modelled country estimate of 77 per 1,000 births (14). This discrepancy appears to have resulted from differences in rates of stillbirth in our study (31/1,000 births) compared to the WHO estimate (42/1,000 births). The rates of stillbirth may have varied because the WHO data are based on the modelled estimates for the entire country, including both urban and rural populations. However, one would predict that combining data from rural and urban populations would have yielded a lower estimate than data derived from a rural population only because of an over-representation of factors in rural communities that increase the likelihood of poor health outcomes, e.g. extreme poverty and low rates of maternal literacy (9). Therefore, the reason for the differences between the WHO estimates and our observations remain uncertain.

The END rate in our study (32/1,000 livebirths) was similar to the WHO-modelled estimate (35/1,000 livebirths) (14). Two conflicting estimates of the regional END rates in the DRC have been published. The Annuaire Sanitaire National 2004 for Equateur province estimated END as 32 per 1,000 livebirths (15) while the National System of Health Information (SNIS) reported an estimate of 20 per 1,000 livebirths (16). Our data suggest that the former estimate is more accurate. The latter figure may be lower because it is based solely on routine data collected by health facilities which are infrequent sites of delivery. Many features of Equateur province that might influence the health outcomes are similar to those in other rural areas of the DRC. For example, Equateur has rates of extreme poverty, illiteracy, barriers to access to healthcare, teen pregnancy, and LBW rates that are similar to the rates in the combined rural areas of the DRC (9). Therefore, we believe that our data are highly representative of rates in many rural areas of the DRC.

We performed the analysis of risk factors for perinatal mortality to identify the potentially-modifiable factors. The risk of perinatal death was 12-fold higher for infants born in the breech presentation. A similar increase in risk has been observed in other studies (17-19). In our study, there were almost equal numbers of deaths associated with breech presentation in deliveries occurring at health centres and in the home, and intrapartum asphyxia was the cause of death assigned in all these cases (data not shown). This observation suggests that breech presentation and its sequelae pose significant challenges in both facility and non-facility settings in rural DRC. The WHO recommends that identifying breech presentations during the antenatal period should be a priority, and women with breech presentations should be delivered in facilities with the capability of performing caesarean deliveries (20). Our data suggest that there is opportunity for improvement in the DRC in both timely identification and referral (50% of breech deliveries occurred at home). However, referral to health centres for delivery, given the current capabilities of these facilities in the DRC, is not likely to improve the outcome because they are not suitable sites for caesarean delivery. The outcome of breech presentation might improve if practical barriers to referral to rural hospitals, e.g. poor roads and non-existent transport vehicles, can be overcome; the level of services in these hospitals is increased; and support for the cost of care is provided by someone other than the patient. Correcting these problems will require a substantial investment in the healthcare infrastructure.

Lack of prenatal care increased the risk of perinatal mortality by three-fold. The impact of antenatal care on perinatal mortality has been well-established (21,22). Further research is needed to identify the barriers to antenatal care in rural DRC. Risk also increased among mothers with parity greater than four. Although not a consistent finding among studies, evidence suggests that grand multiparity increases the risk of the poor pregnancy outcomes, including perinatal mortality, in developed countries (23). Whether this risk association exists in developing countries and whether strategies to reduce grand multiparity would be effective in reducing perinatal mortality in the DRC are questions that would require further investigation.

The absence of a male partner in the home was associated with an increased risk of perinatal mortality. Others have also reported this association (24), although not in a hospital-based study in South Kivu province of the DRC (25). Strategies for support of mothers without male partners have been used with success in other countries (26,27). However, the relevance of the association observed in our study, and, therefore, the potential benefit of these strategies in the DRC, are questionable because the frequency of mothers living without partners was so low.

We found that the LBW infants had a greater than 10-fold increased risk of perinatal death compared to infants of normal birthweight. Leach *et al.* (28) and Ngoc *et al*. (29) made a similar observation. Most strategies to reduce neonatal mortality following the birth of an LBW infant rely upon expensive technologies and services. Instituting these strategies in the DRC would not be practical or possible. However, among infants with birthweights of >2,500 g, improved survival in rural, remote areas is possible with targeted, inexpensive and low-technology interventions, such as maternal tetanus immunization, clean delivery and cordcare practices, exclusive breastfeeding, skin-to-skin (kangaroo) care, and recognition and early treatment of infection (30,31). Infants with birthweights of >1,500 g represented 98% of our cohort. Therefore, increased education of all birth attendants in these practices is warranted.

Decreased risk of perinatal mortality was associated with multiple gestation (twin) birth. The reason for this paradoxical risk reduction is not clear but this apparent beneficial association may have resulted from features of our statistical model. The birthweights of about 70% of twins in our cohort were in the lowest birthweight category in our statistical model. In this category, perinatal mortality was higher among singletons compared to twins. It is possible that twins were more likely to be growth-restricted. At lower birthweights, growth-restricted infants are more likely to survive because they are more mature than their weight-matched peers, and survival is more dependent upon maturity than birthweight. We did not adjust for gestational age in our model, which would have accounted for the protective effect of growth restriction because we were not confident that gestational age could be assigned accurately.

In our cohort, the three most common causes of ENDs were asphyxia, LBW/prematurity, and infection. These three causes have been identified as the most common causes in most LMICs but their relative frequency differs among studies. For example, in a household survey in rural Tanzania, the most important causes of perinatal deaths in order of frequency were infections, asphyxia, and LBW/prematurity (32). By contrast, in a retrospective hospital-based study in North Kivu, DRC, LBW was the predominant cause of death (25). The variability in the relative frequency of individual causes may depend upon features of the environment, demography of the population, and the healthcare system. An additional, and perhaps more important, factor influencing the reported causes of death is the methodology used for assigning a cause. In our study, assignment of a cause of END was based on the local physician's knowledge and familiarity with the expected diseases in the region. Assignment rarely included laboratory investigation and never an autopsy. A rigorous, standardized approach to collecting and analyzing historical data, such as verbal autopsy, was not used. Therefore, our data should be interpreted with some caution.

We did not attempt to assign causes of stillbirth. In the absence of laboratory support, or an alternate rigorous methodology for assigning a cause of stillbirth, we felt that the data would be unreliable. Although we did not assign any cause of stillbirths, we differentiated fresh from macerated stillbirth. This differentiation may be critical in developing strategies to reduce perinatal mortality because fresh stillbirths occur proximate to delivery and, are, therefore potentially preventable. By contrast, macerated stillbirths usually occur more than 12 hours before delivery and less likely to be preventable (33). In our cohort, approximately two-thirds of the stillbirths were fresh. This is in contrast to some previous studies in resource-poor settings where the rate of macerated stillbirth exceeded the rate of fresh stillbirth. However, these studies were often based on observations in hospital-based populations, where intrapartum foetal deaths may have been prevented, or on mathematical modelling (34). Recent literature suggests that community-based stillbirths are primarily fresh rather than macerated (7,35,36). Although misclassification of stillbirth may have occurred in our study, a major emphasis during the preparation of birth attendants for the study was proper differentiation between fresh and macerated stillbirths. In addition, in a setting in which caesarean deliveries were virtually unavailable, one would expect a high rate of intrapartum death. Therefore, we are confident that foetal death proximate to delivery is a significant and potentially-modifiable contributor to perinatal mortality in the DRC.

Over 50% of the ENDs occurred during the day following birth, and an additional 7% occurred during the next day. This finding is similar to observations in a rural population in India (37). Therefore, the most effective strategies to reduce mortality will be those that treat the causes of early mortality. One strategy that may have an impact on one cause of neonatal death—birth asphyxia—is the promotion of more effective resuscitation at the time of birth (38).

### Conclusions

Based on the findings of the study, we conclude that perinatal mortality is a significant problem in rural DRC, with approximately one-half of these deaths occurring in the foetus. Our identification of specific factors that increase risk does not prove a cause-and-effect relationship between the factor and mortality. However, potential strategies for reducing perinatal mortality might include reducing exposure to the factors, for example, by reducing the incidence of higher-order multiparity, increasing enrollment in antenatal care, and improving the management of birth asphyxia. Reducing neonatal mortality associated with breech presentation and intrapartum stillbirth would require identification and timely referral of mothers with foetuses in the breech position and other high-risk conditions. However, this strategy would only be effective if barriers to referral could be eliminated, and the capabilities of rural hospitals could be increased.

## ACKNOWLEDGEMENTS

The authors gratefully acknowledge the contributions of the First Breath DRC Study Group, which included many physicians, nurses and nurse-midwives, traditional birth attendants, and community health workers from the medical districts of Karawa, Tandala, Loko, and Bogose Nubea in Equateur province. The authors also appreciate the contributions of mothers who voluntarily consented to participate in the study.
